# Prioritizing river basins for intensive monitoring and assessment by the US Geological Survey

**DOI:** 10.1007/s10661-020-08403-1

**Published:** 2020-06-27

**Authors:** Peter C. Van Metre, Sharon Qi, Jeffrey Deacon, Cheryl Dieter, Jessica M. Driscoll, Michael Fienen, Terry Kenney, Patrick Lambert, David Lesmes, Christopher A. Mason, Anke Mueller-Solger, Marylynn Musgrove, Jaime Painter, Donald Rosenberry, Lori Sprague, Anthony J. Tesoriero, Lisamarie Windham-Myers, David Wolock

**Affiliations:** 1 US Geological Survey, Austin, TX USA; 2 US Geological Survey, Portland, OR USA; 3 US Geological Survey, Richmond, VA USA; 4 US Geological Survey, Baltimore, MD USA; 5 US Geological Survey, Lakewood, CO USA; 6 US Geological Survey, Madison, WI USA; 7 US Geological Survey, West Valley City, UT USA; 8 US Geological Survey, Reston, VA USA; 9 US Geological Survey, Sacramento, CA USA; 10 US Geological Survey, Norcross, GA USA; 11 US Geological Survey, Menlo Park, CA USA; 12 US Geological Survey, Lawrence, KS USA

**Keywords:** Monitoring design, Federal research, Hydrology, Basin selection

## Abstract

The US Geological Survey (USGS) is currently (2020) integrating its water science programs to better address the nation’s greatest water resource challenges now and into the future. This integration will rely, in part, on data from 10 or more intensively monitored river basins from across the USA. A team of USGS scientists was convened to develop a systematic, quantitative approach to prioritize candidate basins for this monitoring investment to ensure that, as a group, the 10 basins will support the assessment and forecasting objectives of the major USGS water science programs. Candidate basins were the level-4 hydrologic units (HUC04) with some of the smaller HUC04s being combined; median candidate-basin area is 46,600 km^2^. Candidate basins for the contiguous United States (CONUS) were grouped into 18 hydrologic regions. Ten geospatial variables representing land use, climate change, water use, water-balance components, streamflow alteration, fire risk, and ecosystem sensitivity were selected to rank candidate basins within each of the 18 hydrologic regions. The two highest ranking candidate basins in each of the 18 regions were identified as finalists for selection as “Integrated Water Science Basins”; final selection will consider input from a variety of stakeholders. The regional framework, with only one basin selected per region, ensures that as a group, the basins represent the range in major drivers of the hydrologic cycle. Ranking within each region, primarily based on anthropogenic stressors of water resources, ensures that settings representing important water-resource challenges for the nation will be studied.

**Electronic supplementary material:**

The online version of this article (10.1007/s10661-020-08403-1) contains supplementary material, which is available to authorized users.

## Introduction

The mission of the US Geological Survey (USGS) is to provide data and unbiased science on the natural resources of the country. In 2007, the USGS published a Science Strategy (USGS 2007) that included a science direction of “A Water Census of the United States—Quantifying, Forecasting, and Securing Freshwater for America’s Future”. In 2013, the USGS outlined a strategy for a national census of water availability built around *observing*, *understanding*, *predicting*, and *delivering* water science to meet the nation’s water-resource needs (Evenson et al. 2013). During 2018 to 2020, the USGS Water Resources Mission Area (WMA) has been designing and implementing new integrated water-science programs to meet the objectives of an integrated national assessment of water availability. A critical aspect of implementing these programs is the development of a scientifically defensible study design at the national scale.

Programs tasked with assessing natural resources for regional to continental domains face many challenges including developing scientifically defensible monitoring designs, identifying and accessing representative sampling locations, and generating the data necessary for broad characterization, process understanding, and modeling. Hydrologic monitoring network design has received considerable attention in the scientific literature, although mostly regarding site selection, sampling timing, constituents measured, and methods (Bartram and Ballance 1996; Strobl and Robillard 2008; Telci et al. 2009; Fienen et al. 2010; de Souza Fraga et al. 2019; Nguyen et al. 2019). Little has been published, however, that addresses broad questions of scope and design for national-scale assessments, such as the selection of multiple river basins, aquifers, or regions for study.

Two Federal agencies—the US Environmental Protection Agency (EPA) and the USGS—have developed and applied different national-scale designs for water resources assessments in recent decades. The EPA National Aquatic Resource Surveys has conducted assessments by sampling sites nationwide selected using a probabilistic approach (EPA 2016). This approach is well suited for estimating the environmental conditions across regions as well as the USA. The USGS National Water Quality Assessment (NAWQA) Project groundwater assessments have selected wells using stratified random designs (Belitz et al. 2003; Belitz et al. 2010) to characterize major aquifers (Bexfield et al. 2019), similar in some respects to the EPA approach.

An alternative approach for large-scale assessment is to collect data more intensively (e.g., spatially, temporally, parametrically) in sub-regions within the larger domain to facilitate process analysis and support development of more complex models. In 1991, the USGS launched the NAWQA Project using such a design with 60 mid-sized “study units” (generally about 20,000 to 60,000 km^2^) distributed across the USA and studied over the following decade (Leahy et al., 1990). Study units were selected to achieve coverage of major hydrologic regions, important agricultural areas and population centers, and with consideration of water quality concerns. A similar approach was used by the NAWQA Regional Stream Quality Assessments from 2013 to 2017 which selected five large ecoregions for intensive study; regions were selected with intensive agricultural and urban land uses and to represent diverse hydrologic settings of the contiguous United States (CONUS) (May et al. 2020). These efforts helped to inform the development of the method presented herein for prioritizing basins for assessment by the USGS.

Three new USGS WMA programs instrumental in launching this basin selection effort are NextGen Water Observing Systems (NGWOS), Integrated Water Availability Assessments (IWAAs), and Integrated Water Prediction (IWP). The objectives of NGWOS are to advance the technologies of monitoring and data delivery and generate data in support of water resource assessments (e.g., IWAAs) and advanced modeling of hydrologic systems (e.g., IWP) (Eberts et al. 2019). The new USGS programs will utilize a design that combines intensive monitoring and assessment in Integrated Water Science Basins (referred to hereafter as, “priority basins”) with state and national monitoring programs operated by the USGS and others. The priority basins are designed to support two major objectives: address high-priority water resource issues and support advancements in hydrologic modeling. To address important water resource challenges, the priority basins need to represent a broad range of high-priority socio/economic and environmental/climatic issues facing the nation (Evenson et al. 2013). To advance hydrologic modeling, the priority basins should include settings where a variety of important hydrologic processes are represented. Thus, diversity in temperature, precipitation, elevation, and other major drivers of the hydrologic cycle are important considerations in basin selection, as well as the many physical and chemical alterations of hydrologic systems by human actions. Data collected in the priority basins, combined with enhancements of existing USGS monitoring networks across the country and external (to USGS) data, should facilitate the knowledge transfer needed to inform modeling and decision making at multiple scales across the USA.

Given that substantial public resources are to be spent over the next decade on the priority basins, a systematic, scientifically defensible approach was needed to guide the ranking and selection of basins for study. In spring 2019, the WMA established a Basin Selection Team (BST) composed of 15 USGS scientists with varied backgrounds and from different parts of the country, all of whom are among the authors of this article. The BST was charged with developing basin selection criteria and implementing a systematic, quantitative approach for ranking basins for study. The goal in developing such an approach was to create a relatively short list of candidate basins, all of which should be reasonable choices for intensive monitoring and assessment based on the objectives of the USGS programs. Creating this list is the first step in the basin selection process. Many considerations in selecting areas for long-term intensive study are subjective, such as the importance of local issues identified by stakeholders. Thus, the second step in basin selection includes stakeholder input including feedback from the USGS Water Science Centers and other USGS Mission Areas, other Federal agencies (e.g., EPA, US Bureau of Reclamation, and US Fish and Wildlife Service), State and local environmental management agencies, and various non-governmental organizations with interests in water resources. Final selection of priority basins will be made by WMA management from the short list of candidate basins developed herein and from stakeholder input.

## Approach

The numerical-ranking approach first divided the CONUS into 18 hydrologic regions wherein homogeneity of major hydrologic drivers and processes within each region was maximized and heterogeneity among the regions was maximized. A list of 163 candidate basins was developed based on the 203 level-4 hydrologic units (HUC04) (https://water.usgs.gov/GIS/huc.html) covering the CONUS with some of the smaller HUC04s combined to form candidate basins (Online Resource Table S-1). Although the HUC system and candidate basins are developed on the stream network, USGS monitoring will include groundwater, other surface waters (lakes, reservoirs, wetlands, and estuaries), and other aspects of the hydrologic cycle (e.g., evapotranspiration, snowpack, soil moisture). Furthermore, the boundaries of the priority basins will be flexible during study implementation to allow for a more complete assessment of the variety of water resource factors and settings in the area. Given the unique environmental settings of Hawaii and Alaska relative to the CONUS, it is unlikely that any watersheds in those two states will be selected as one of the first 10 priority basins. However, because improving water prediction in those settings is important, prioritizing those areas after the initial 10 priority basins are implemented will be considered should funding continue to be available. From a potential list of several hundred watershed characteristics, 10 geospatial variables were selected to rank candidate basins within each of the hydrologic regions.

### Criteria for ranking

Three criteria were identified to be considered in the numerical ranking of basins, and several qualitative factors were identified that could be considered for final selection of priority basins. The three criteria for numerical ranking and their rationale are the following:

#### Natural factors

Select basins to represent diverse terrain and hydrologic factors governing the hydrologic cycle. This criterion addresses the development and improvement of hydrologic models, by attempting to select basins that, in combination, represent the range in important natural hydrologic settings in the CONUS. Importance was judged based on concepts described by (Wolock et al. 2004; Markstrom et al. 2016; Stanislawski et al. 2018).

#### Anthropogenic factors

Prioritize regions with large areas and proportions of stream kilometers and(or) aquifers affected by anthropogenic stressors (land use, climate change, and water use) and with potentially or historically rapid changes in stressors of water availability. Selecting basins with high levels of anthropogenic stress on their water resources helps ensure that the USGS studies will focus on some of the most important water resource challenges of the nation.

#### Importance of resource to receptors

Prioritize basins based on the importance of water resources provided within and outside of the basin for human uses and ecological needs. This criterion focuses on the overall water balance of the basin including the importance of water leaving the basin to downstream users, the extent of water availability concerns and risks, and important natural dependencies on the hydrologic system, for example the numbers of at-risk aquatic species.

Once the field of candidate basins has been narrowed by numerical ranking, several other factors could be considered with input from stakeholders. These include the extent of current and historical monitoring and modeling, settings and issues representing critical monitoring needs, and pressing national water resource issues. Priority basins are envisioned to be state-of-the-art environmental monitoring and assessment research test beds. Thus, establishing priority basins where monitoring is currently and historically extensive is logical. Intensive integrated monitoring could potentially address complex issues that are critical to a basin, region, or the country. Effects of climate change on water resources, loss of aquatic species and ecosystem functions, and spread of harmful algal blooms are examples of critical monitoring needs and pressing national water resource issues that can be addressed by USGS programs.

### Candidate basins and regional framework

Two levels of spatial organization were used in the basin selection process: candidate basins and hydrologic regions. During early WMA planning of the new programs, it was decided that basins of about 25,000 to 50,000 km^2^ (about 10,000 to 20,000 mi^2^) would be considered for intensive monitoring. This basin size is similar to that of the Delaware River Basin—the pilot Integrated Water Science Basin—and approximately corresponds to the size of a HUC04. Importantly, HUC04s, with some smaller basins combined, were the basis of the original NAWQA Project study units in the 1990s (Leahy et al., 1990). The BST followed a similar initial approach as the NAWQA Project, starting with HUC04 polygons and using professional judgement to combine some small contiguous HUC04s to create candidate basins similar to the target size identified by the new programs. The 203 HUC04s in the CONUS were thus reduced in number to 163 candidate basins with a median basin area of 46,600 km^2^ (Online Resource Table S-1). Candidate basins were clipped at international boundaries and at the US coastline.

Establishing a regional framework based on hydrologic characteristics and distributing basins across the CONUS with no more than one selected per hydrologic region will help ensure that each basin represents a unique combination of important natural characteristics (criterion 1). The BST considered four regional frameworks: EPA ecoregions used for the CONUS by the National Rivers and Streams Assessment (EPA 2016), physiographic regimes classified based on hydrologic factors (Stanislawski et al. 2018), water resource regions (the largest scale HUCs), and hydrologic landscape regions (HLRs) (Wolock et al. 2004). Based on discussions within the BST and statistical and spatial data analyses, HLRs were chosen as the basis of the regional framework. The BST determined that HLRs well represent differences in the major drivers of the hydrologic cycle relevant at the HUC04 level.

**Fig. 1:**
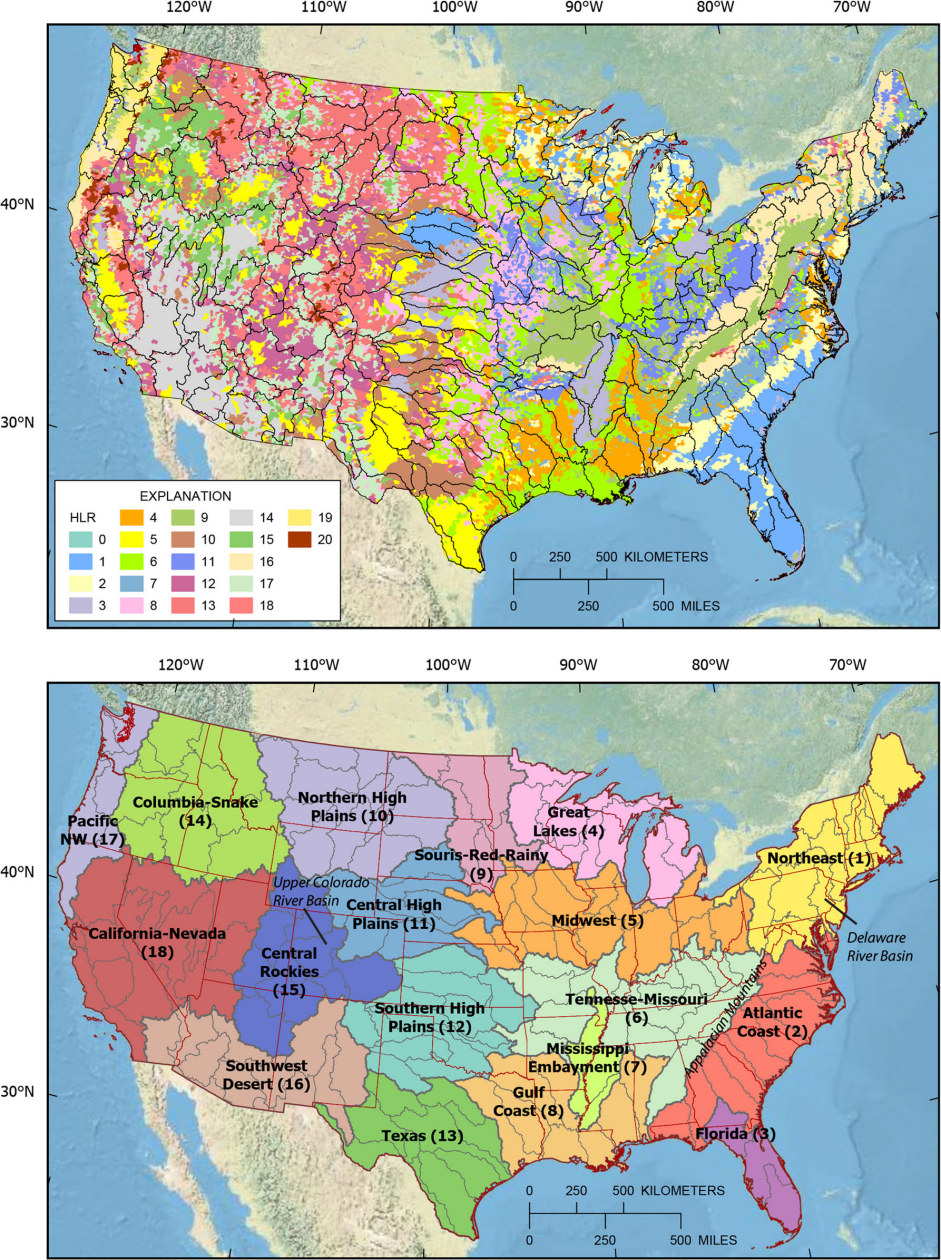
The 163 candidate basins overlain with the hydrologic landscape regions (HLR; Wolock et al. 2004) (upper map; HLRs are described in Online Resource Table S-2) and the 18 hydrologic regions (lower map; the number in parenthesis after region name is used in subsequent figures). Place names used in the text are shown in italic font

HLRs, like ecoregions and physiographic regimes, do not share boundaries with the candidate basins; thus, each candidate basin can overlap with multiple HLRs. Some logical way of grouping candidate basins relative to the HLRs was needed. The method chosen was to overlay candidate basins with the 20 HLRs covering the CONUS and determine the fraction of the candidate basin represented by each HLR (Fig. 1, upper map). The candidate basins were grouped into clusters using k-means clustering as implemented in scikit-learn (Pedregosa et al. 2011) and, based on HLR proportions and the coordinates of the centroid of each candidate basin, the basins were combined into 18 hydrologic regions (Fig. 1, lower map). Inclusion of the centroid location forced the delineation of contiguous clusters, each cluster representing a group of candidate basins with a similar mixture of HLRs.

One example illustrating the clustering process is the Atlantic Coast hydrologic region. A series of roughly parallel river basins, and therefore HUC04-based candidate basins, drain the Appalachian Mountains flowing southeast to the Atlantic Ocean and crossing several HLRs (Fig. 1). Each of these candidate basins has a relatively similar mixture of HLRs reflecting the natural characteristics of the southeastern United States, and they are different from the mixtures of HLRs that make up the other hydrologic regions.

**Fig. 2:**
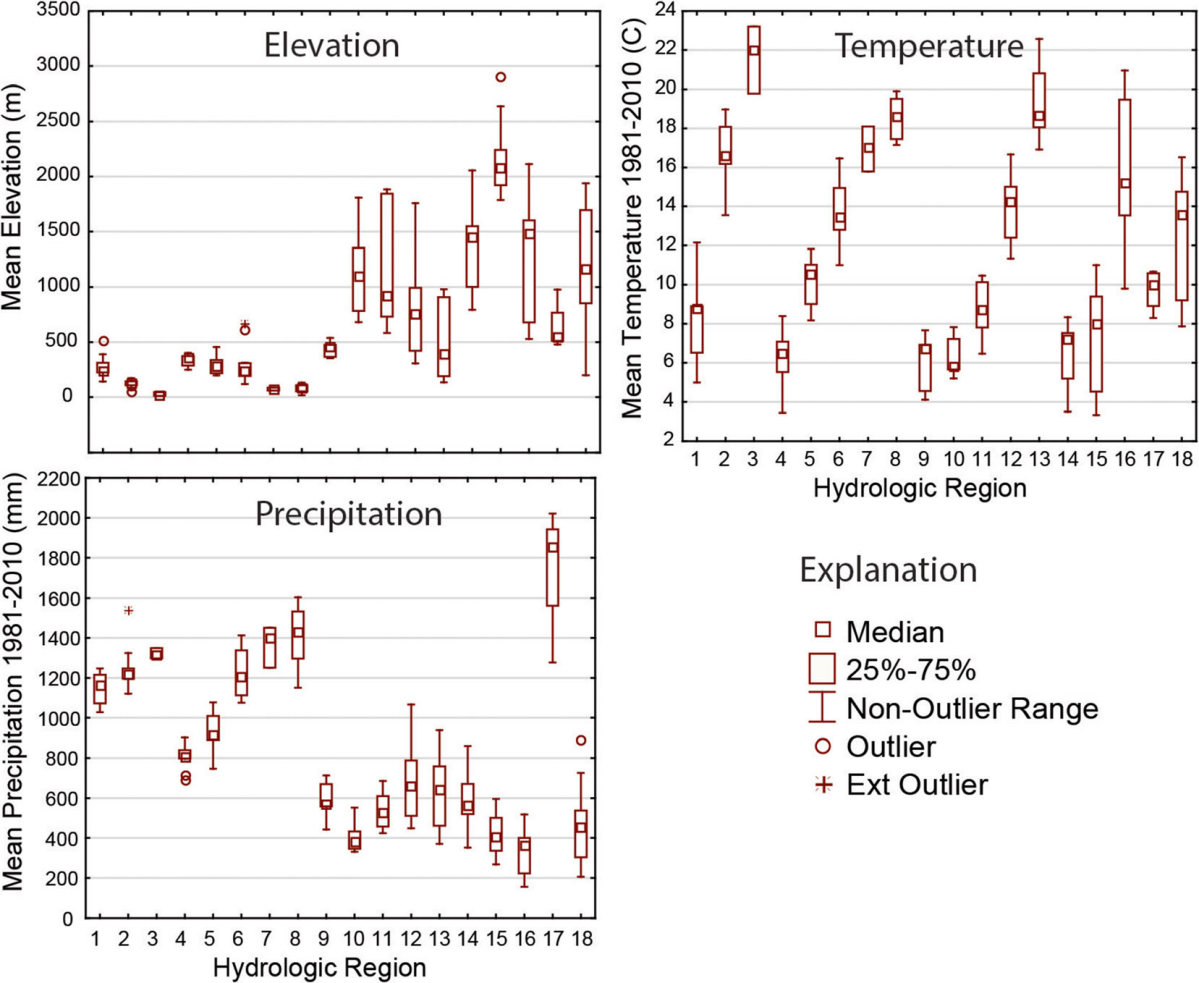
Box plots showing the distribution of three variables within candidate basins in each of the 18 hydrologic regions. Each box comprises the mean values of the variable for the candidate basins in the region. See Fig. 1 for names of hydrologic region numbers

The variability in three fundamental characteristics that affect the hydrologic cycle—precipitation, temperature, and elevation—shows clear patterns among the 18 hydrologic regions (Fig. 2). The regions are numbered for these graphs from north to south and east to west (Fig. 1). Elevation is clearly higher in the west and lower in the east, as indicated by comparing the nine regions roughly east of the High Plains to the nine regions in the West. With the exception of the Pacific Northwest (region 17), precipitation is higher in the east and lower in the west. Temperature has a repetitious north-to-south pattern for regions in the eastern, central, and western CONUS. One objective in creating these hydrologic regions is to create groupings of basins that are relatively homogeneous in terms of the major drivers of the hydrologic cycle and relatively unique from the other regions. Figure 2 suggests this objective has been met in that the 25th to 75th quartiles indicated by the box plots are relatively short compared with the overall scale of each graph, and each region has a somewhat unique combination of the three characteristics.

### Ranking candidate basins within regions

A wide variety of geospatial variables is available digitally at the national (or CONUS) scale (e.g., Falcone et al. 2019; Falcone 2018). Approximately 300 variables were compiled in a Geographic Information System (GIS) for consideration in ranking. Many of the ~300 variables were eliminated by assuming that the hydrologic regions were adequately representing the natural factors described for criterion 1. Given the many variables representing anthropogenic activities and environmental stressors that are available, the BST strived for parsimony in selecting variables that represent important features and are relatively independent of each other. There are, for example, many land-use class variables available from multiple sources and dates which were condensed into two major classes: urban and agriculture. Measures of decadal change in land use were considered, but urban land use and change in urban land use were highly correlated. Based on professional judgement, 10 variables were selected for ranking basins within each of the 18 hydrologic regions (Table 1).

**Table 1:** Variables used in numerical ranking of candidate basins

Variable name	Description	Source
Urban	Mean total area of urban land use in 2012 (%)	(Falcone et al. 2019)
Ag	Mean total area of agricultural land use in 2012 (%)	(Falcone et al. 2019)
Eco_sensitivity	Number of at-risk aquatic species (mean) and area of rare ecosystems (%)	https://www.epa.gov/wsio
PPT_change	Mean projected change in precipitation to 2070–2099 (mm)	https://climate.northwestknowledge.net/MACA/
Tot_WU	Total freshwater withdrawals (million gallons per day (mgd))	(Dieter et al. 2018)
Runoff	Runoff generated in the basin (mgd)	(McCabe and Wolock 2011)
WU_runoff	Ratio of Tot_WU:Runoff (unitless)	Calculated from (McCabe and Wolock 2011)
GW_sto_change	Mean rate of change in GW storage (cm/y)	(Velpuri et al. 2019)
ResVol	US Army Corps of Engineers: reservoir storage volume per area (m3/km2)	https://nid.sec.usace.army.mil/
FireHzd	Mean USDA Forest Service: fire hazard score (unitless)	https://www.firelab.org/project/wildfire-hazard-potential

**Fig. 3:**
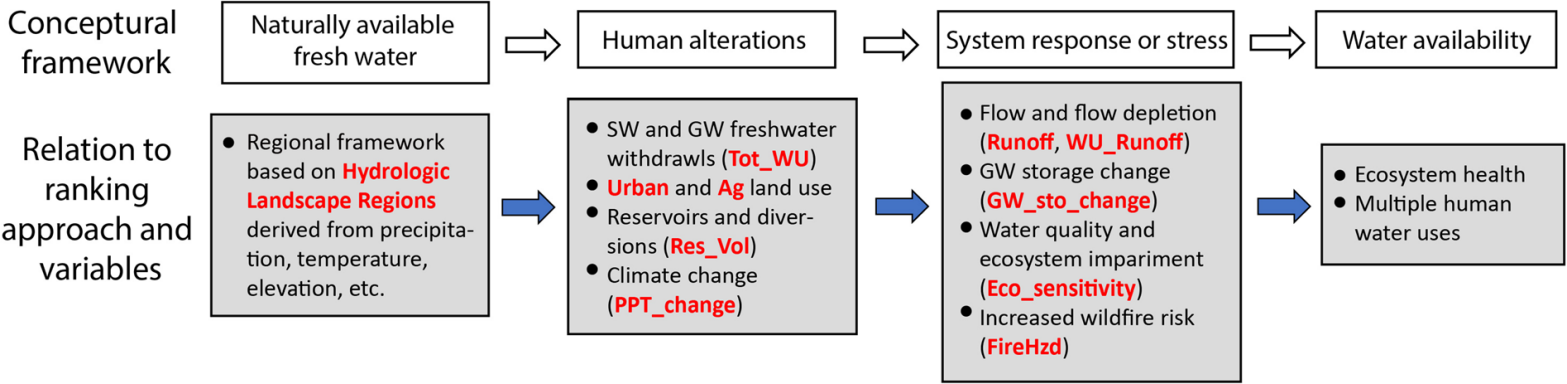
Conceptual framework of major factors affecting water availability in relation to the ranking approach and variables

A simple conceptual framework of the major factors affecting water availability and our attempt to capture the key aspects of that framework in the ranking process are shown in Fig. 3. Water availability begins with the natural drivers of the hydrologic cycle. Those natural drivers—precipitation, temperature, elevation, and others—were used to develop the HLRs. Natural drivers are factored into ranking by grouping the candidate basins into hydrologic regions and limiting selection to one basin in any region. There are many human activities that affect water availability by altering the hydrologic cycle (e.g., withdrawals from streams and aquifers, constructing dams, changing the climate) or altering water quality (e.g., runoff and discharges from urban and agricultural lands). Hydrologic systems respond to these natural and human drivers with changes in water quantity and quality which can affect availability for human uses and stress ecosystems. These stressors and responses are represented by the 10 ranking variables.

No comprehensive national water quality metric is available that takes into account the variety of chemical and elemental threats to water supplies and ecosystems; however, urban and agricultural land use are strongly associated with occurrence of contaminants in streams and shallow aquifers and with ecological impairments (e.g., Allan 2004; Coles et al. 2012; Nowell et al. 2018; Waite et al. 2019; Van Metre et al. 2019), thus land use accounts indirectly for these system responses. The ecosystem sensitivity variable factors in measures of the current status of stream ecosystems representing a system response. It could be debated where some of the ranking variables fit in this framework, but we contend that all are relevant to water availability and that, as a group, they reasonably represent the most important factors affecting availability in a parsimonious way.

The rationale for choices of ranking variables is given here by category with the variable names from Table 1 in *italic* font and, for clarity, when referred to in subsequent text. These 10 and other selected GIS variables are provided in Online Resource Table S-3, and variable definitions are given in Online Resource Table S-4.

- Urban land use—total area of urban land use, *Urban*, correlates strongly to population and use of public water supplies, both of which exert a strong influence on water availability. Urban land use also has a strong negative effect on water quality for downstream human uses and ecosystem health (Allan 2004; Coles et al. 2012) and, in some cases, on shallow groundwater (DeSimone et al., 2015); thus, it is an indicator of anthropogenic stress. Major stressors associated with urban land use include streamflow alteration; stream and lake eutrophication from shallow groundwater, runoff, and point-source discharges; elevated pesticides; and various inorganic and organic contaminants (Paul and Meyer 2001; DeSimone et al. 2015; Moore et al. 2003; Coles et al. 2012; Waite et al. 2019).
- Agricultural land use—total area of agriculture (the sum of cropland and pasture), *Ag*, also is a measure of anthropogenic stress. Agricultural land use has a much larger footprint than urban land use nationally, is the largest consumptive user of water, alters the hydrologic cycle in many ways, and introduces a wide range of nutrients and contaminants to streams and aquifers (Böehlke 2002; Burkart and Stoner 2008; Gentry et al. 2009; Capel et al. 2018; Nowell et al. 2018; Schmidt et al. 2019).
- Ecosystem sensitivity—Stream ecosystems provide essential services to the organisms living in them and to the benefit of humans (Millennium Ecosystem Assessment 2005); however, representing ecosystem services in a single variable is difficult. We chose instead to combine five ecological indicator variables provided by the EPA Watershed Index Online (WSIO) that capture the “ecosystem sensitivity” of each candidate basin: counts of at-risk plant and animal species for aquatic and wetland environments (4 variables) and the percent rare ecosystem of the watershed. These variables apply to the candidate basin but do not capture potentially important downstream ecological needs such as those associated with estuaries and river deltas. The WSIO provides these variables at the HUC12 scale (small catchments nested within HUC04s). The means of each of the four at-risk-species counts for HUC12s in each candidate basin were summed and that sum was ranked across all candidate basins. Those ranks and the ranks of the percent of area identified as rare ecosystem in each candidate basin were converted to percentiles (scaled from 0 to 1), summed and divided by 2 to scale the resulting *Eco_Sensitivity* as a single ranking variable.
- Climate change—*PPT_change* represents areas where increases or decreases in precipitation are projected. This is an important term to help prioritize basins because either increase or decrease in precipitation can substantially alter the hydrologic cycle (Trenberth 2011). *PPT_change* was developed from mean estimates of 20 global climate models downscaled for the CONUS. The specific precipitation term was mean precipitation percent change for 2070–2099 relative to the 1971–2000 mean based on the RCP 8.5 emissions scenario. Source data are available from the Multivariate Adaptive Constructed Analogs (MACA; https://climate.northwestknowledge.net/MACA/).
- Water use—Total freshwater withdrawals, *Tot_WU*, is included as the indicator of total water use demand. Freshwater withdrawals not only represent water extracted for various human uses, they can also have a profound effect on the ecological health of streams (Carlisle et al. 2019). Other metrics were considered (for example, separate values for groundwater and surface water withdrawals, consumptive use, and public supply withdrawals) and are available for additional analysis and decision-making depending on the question (Online Resource Table S-3). Note that this variable is total freshwater withdrawals, which is not necessarily the water use in the basin. This is an important distinction, especially in the west where inter-basin transfers occur as water is withdrawn in one basin and transported and used in other basins. Thus, *Tot_WU* represents the stress caused by freshwater withdrawals at the location of withdrawal, which potentially encompasses inter-basin transfers. Saline withdrawals were not considered in this factor because the BST reasoned that (a) the primary withdrawals of saline water are for power plant cooling, and consumptive use of those saline withdrawals is small; and (b) the saline withdrawals are primarily surface water from estuaries, and coastal environments where the impacts on fresh water availability are probably minor.
- Runoff—*Runoff* is the estimated water balance model amount of runoff produced within each candidate basin. *Runoff* is the flow per unit area delivered from each 4 × 4-km grid cell to streams and rivers in units of millimeters per month (McCabe and Wolock 2011), summed for each candidate basin. Thus, it broadly represents the amount of water available from local sources—surface runoff and baseflow—for use including instream flows for ecosystems. It also is a representation of the importance of the basin in producing water for downstream uses. The headwaters of the Colorado and Gunnison River Basin (hereafter, Colorado–Gunnison (Online Resource Table S-1)) is a good example of a basin providing essential water to downstream users.
- Water demand stress—Two variables were included to represent water demand stress: the ratio of *Tot_WU* to *Runoff* (*WU_runoff*) and the rate of change in ground water storage (*GW_sto_change*). Basins with high *WU_runoff* ratios generally are under high water-use stress, with local freshwater withdrawals similar to or exceeding locally generated runoff. High *WU_runoff* ratios are found in arid settings with high water use demand such as the Lower Gila (1507) and Southern Mohave–Salton Sea (1810), both of which have *WU_runoff* of about 10. *GW_sto_change* was modeled by (Velpuri et al. 2019) using the Gravity Recovery and Climate Experiment (GRACE) data set. This variable prioritizes areas where large changes in groundwater storage (and groundwater levels) in either the positive or negative direction have occurred. In some cases, these changes have impacted and(or) been caused by changes to the surface water system, for example, in the California Central Valley where recent increases in groundwater pumpage have occurred in response to decreased availability of surface water (Famiglietti 2014).
- Flow alteration—Streamflow alteration caused by a variety of anthropogenic actions and climate change impact water availability and ecosystem health (Carlisle et al. 2011; Carlisle et al. 2019). Reservoirs and accompanying diversions are a major cause of streamflow alteration. *ResVol* is the area-normalized volume of reservoir storage at normal pool elevation and is used here as an indicator of flow alteration.
- Fire hazard—Wildfires can substantially alter the hydrologic cycle and quality of streams, especially in the semi-arid west (Smith et al. 2011). *FireHzd* is a map layer representing wildfire hazard potential developed by the US Forest Service, Fire Modeling Institute. This data layer is designed to “depict the relative potential for wildfire that would be difficult for suppression resources to contain”.

Each of the 10 variables, represented as raster or polygon geospatial data, was overlain on either HUC10s (small watersheds nested within HUC04s) or the candidate basins or, in the case of the variables used to compute *Eco_sensitivity*, were available at the HUC12 scale. Variables were averaged (mean value) across the candidate basin (e.g., *Urban*) or summed across the candidate basin (e.g., *Tot_WU*), as appropriate (Table 1). Each variable associated with the 163 candidate basins was then percentile ranked for the CONUS (ranked then the rank divided by 163 to compute the percentile of the rank of the variable scaled from 0 to 1). This step was taken to adjust for different units among variables and to avoid undue influence from outliers.

A weighting factor of 2 was applied to four variables representing the importance of water resources in the region and for downstream uses: *Tot_WU*, *Runoff*, *WU_runoff* ratio, and *GW_sto_change*. The percentile ranks for these variables were multiplied by 2 to double their importance relative to the other six variables. Finally, the ranks were summed, and basins were ordered by total score within each hydrologic region. The percentile-ranked and weighted variables, summed scores, and regional ranks for all basins are given in Online Resource Table S-5.

The selection of variables and the manipulations of those variables in the ranking process influence the outcome. Each of the variables selected was assumed to be relatively independent of the others. Each, by the very nature of the approach of summing them to calculate scores used in ranking, has some influence on the resulting total score for candidate basins with the four variables given a double weight having more influence. The relations of variables and scores to ranks, however, is complicated by grouping the sites by hydrologic region and ranking within each region. This is because the range in composite scores varies substantially between regions.

Many of the 10 variables correlate significantly to each other (Table 2), which is partly a consequence of having a large sample size of 163 basins. The highest correlations among variables are for *Urban* and *Runoff* and for *Ag* and *FireHzd*. The former is because more people tend to live in the wetter parts of the country and the latter because wildfires tend to occur on undeveloped lands. None of these correlations were thought to be a problem in using the 10 variables in ranking.

**Table 2:** Pearson’s correlation matrix for the 10 percentile-ranked variables

	Urban	Ag	ResVol	Fire Hzd	PPT change.	Tot_WU	WU_Runoff	Runoff	GW_sto change	Eco sensitivity
Urban	1.00	*0.22*	*0.33*	−0.14	*0.53*	*0.46*	*−0.23*	*0.66*	−0.12	*0.33*
Ag	*0.22*	1.00	−0.12	*−0.65*	*0.19*	0.14	−0.09	*0.20*	*0.20*	*−0.35*
ResVol	*0.33*	−0.12	1.00	*0.16*	*0.19*	*0.28*	*−0.17*	*0.44*	−0.05	*0.29*
FireHzd	−0.14	*−0.65*	*0.16*	1.00	*−0.37*	0.08	0.15	−0.11	−0.05	*0.51*
PPT_change	*0.53*	*0.19*	*0.19*	*−0.37*	1.00	0.14	−0.28	0.43	−0.11	−0.01
Tot_WU	*0.46*	0.14	*0.28*	0.08	0.14	1.00	0.46	0.48	−0.05	*0.35*
WU_Runoff	*−0.23*	−0.09	*−0.17*	*0.15*	*−0.28*	*0.46*	1.00	−0.51	*0.16*	0.00
Runoff	*0.66*	*0.20*	*0.44*	−0.11	*0.43*	*0.48*	*−0.51*	1.00	*−0.19*	*0.35*
GW_sto_change	−0.12	*0.20*	−0.05	−0.05	−0.11	−0.05	*0.16*	*−0.19*	1.00	0.00
Eco_sensivity	*0.33*	*−0.35*	*0.29*	*0.51*	−0.01	*0.35*	0.00	*0.35*	0.00	1.00

Italicized values significance at *p* < 0.05.

## Results and discussion

**Fig. 4:**
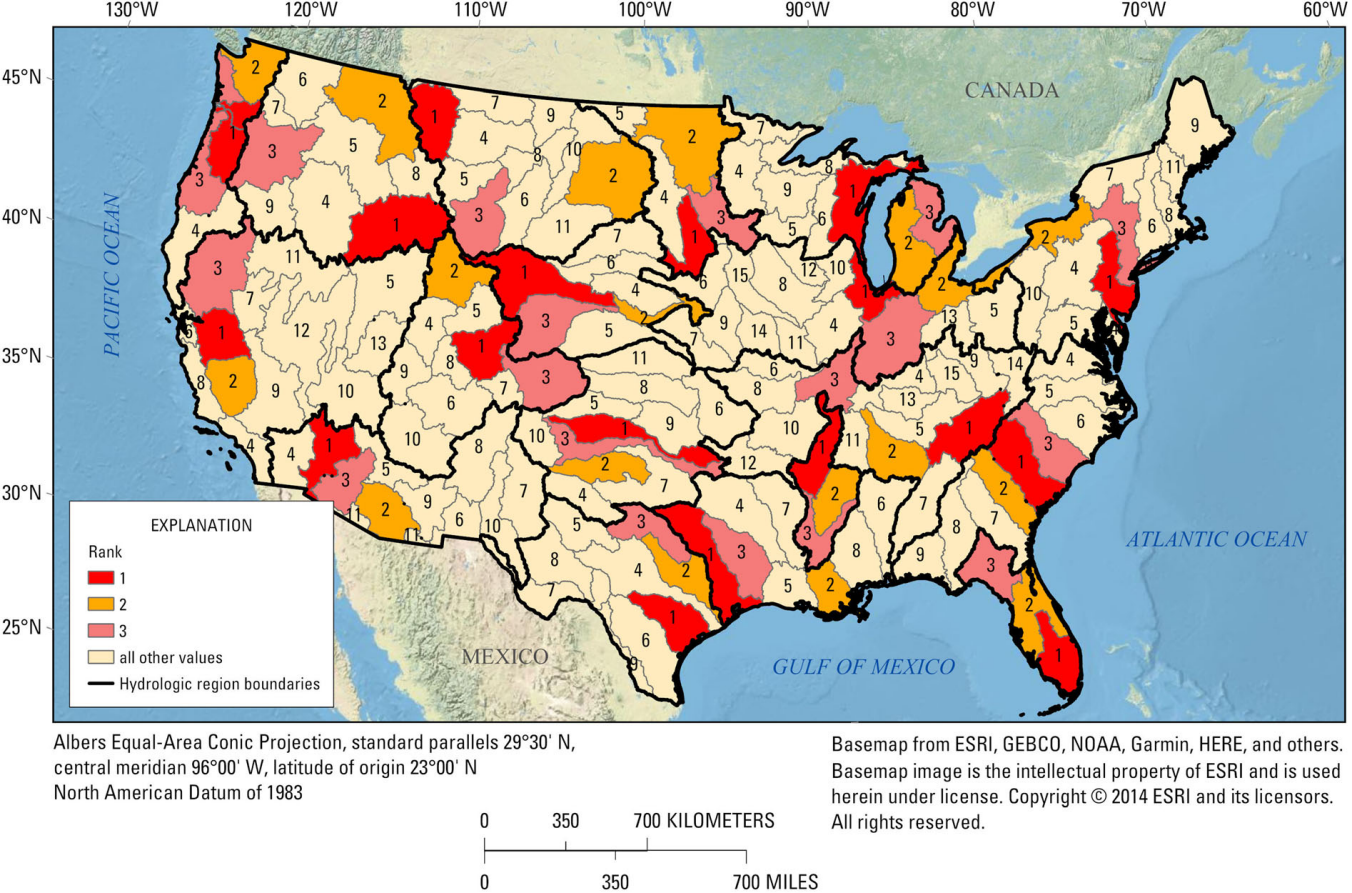
Ranking results for candidate basins within each of 18 hydrologic regions; top-ranked basins are given in Table 2

**Fig. 5:**
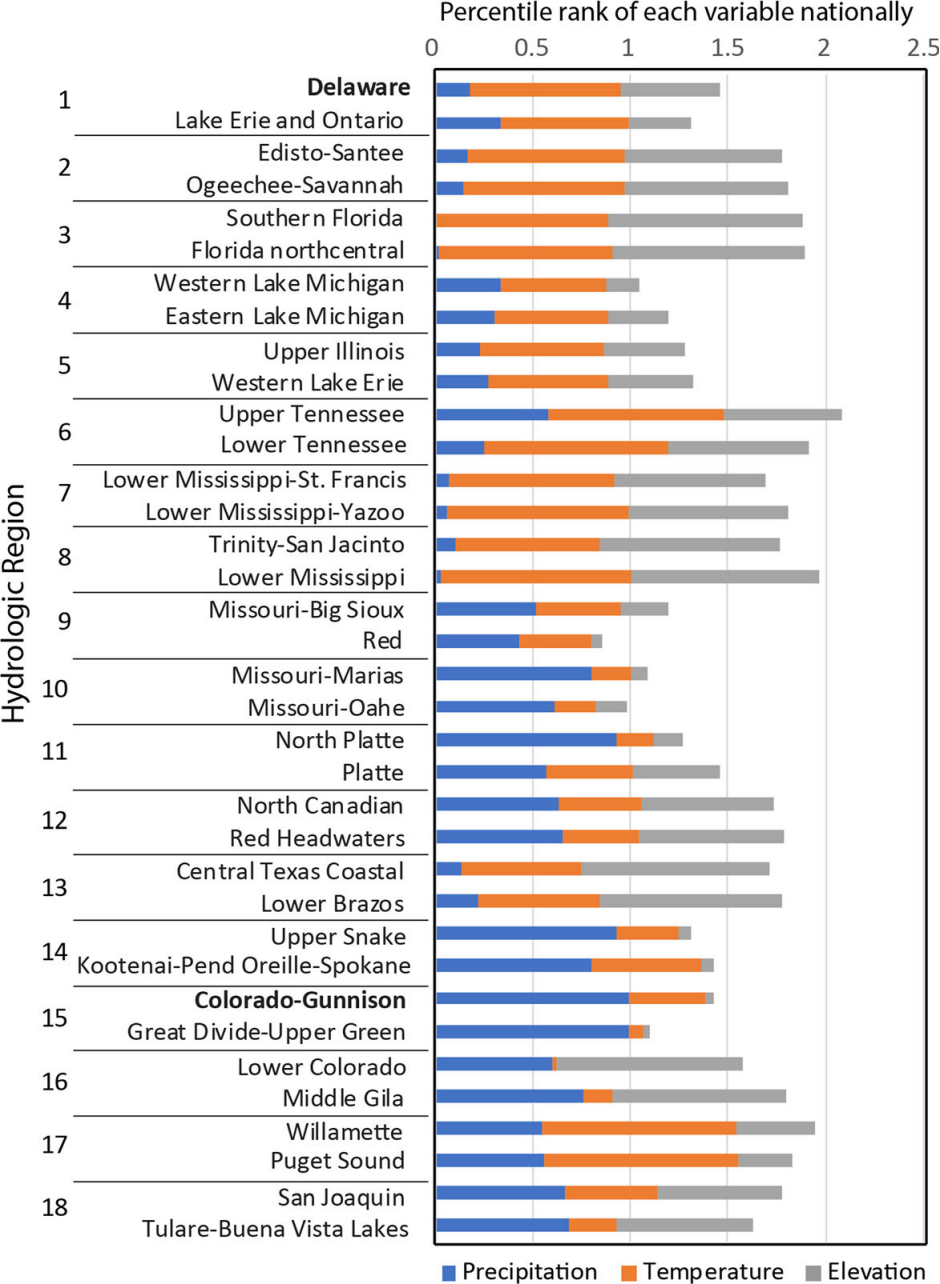
Distribution of 30-year mean precipitation and temperature and mean elevation for the 36 top-ranked basins, with each variable scaled from 0 to 1 based on the range among all basins nationally

The numerical ranking within each hydrologic region was used to reduce the number of candidate basins from 163 to 36, assuming the top 2 candidate basins in each hydrologic region are reasonable choices as priority basins (Fig. 4, Table 3). The 36 basins represent the ranges in major natural factors that affect hydrology across the CONUS, as indicated by the distributions of 30-year mean precipitation and temperature and mean elevation for these basins (Fig. 5). Each variable is scaled from 0 to 1 relative to the national range for candidate basins. Not surprisingly given the approach used, the 36 basins span the range of combinations of these three variables with relatively little overlap (note that the first and second ranked basins from each hydrologic region are similar).

**Table 3:** Top-two candidate basins in each hydrologic region based on numerical ranking

REGION name	Basin ID	Basin name	Region#	Rank
Northeast	204	Delaware	1	1
	411	Lake Erie and Ontario	1	2
Atlantic Coast	305	Edisto–Santee	2	1
	306	Ogeechee–Savannah	2	2
Florida	309	Southern Florida	3	1
	308	Florida northcentral	3	2
Great Lakes	403	Western Lake Michigan	4	1
	405	Eastern Lake Michigan	4	2
Midwest	712	Upper Illinois	5	1
	409	Western Lake Erie	5	2
Tennessee–Missouri	601	Upper Tennessee	6	1
	603	Lower Tennessee	6	2
Mississippi Embayment	802	Lower Mississippi–St. Francis	7	1
	803	Lower Mississippi–Yazoo	7	2
Gulf Coast	1203	Trinity–San Jacinto	8	1
	807	Lower Mississippi	8	2
Souris-Red-Rainy	1017	Missouri–Big Sioux	9	1
	902	Red	9	2
Northern High Plains	1003	Missouri–Marias	10	1
	1013	Missouri–Oahe	10	2
Central High Plains	1018	North Platte	11	1
	1020	Platte	11	2
Southern High Plains	1112	Red Headwaters	12	1
	1109	Lower Canadian	12	2
Texas	1210	Central Texas Coastal	13	1
	1206	Lower Brazos	13	2
Columbia–Snake	1704	Upper Snake	14	1
	1701	Kootenai–Pend Oreille–Spokane	14	2
Central Rockies	1401	Colorado-Gunnison	15	1
	1404	Great Divide–Upper Green	15	2
Southwest Desert	1503	Lower Colorado	16	1
	1505	Middle Gila	16	2
Pacific Northwest	1708	Willamette	17	1
	1711	Puget Sound	17	2
California–Nevada	1804	San Joaquin	18	1
	1803	Tulare–Buena Vista Lakes	18	2

The NGWOS program started a pilot phase in 2018, before the BST was established. The Delaware River Basin was selected in 2018 as a pilot basin for the NGWOS program and, in 2019, as a pilot basin for the IWAAs program. This was the first of the 10 planned Integrated Water Science Basins to be selected in the coming decade as part of the integration of the USGS WMA programs. The Colorado-Gunnison Basin was selected in December 2019 as the second of the 10 priority basins. That selection was made based on the numerical ranking described herein and input from USGS Water Science Centers and regional staff in the west, as well as the US Bureau of Reclamation, the Interstate Council on Water Policy, and the Western States Water Council. Long-term drought conditions, interstate ramifications of the drought, water quality issues, stakeholder support, and alignment with Department of the Interior and USGS priorities make the Colorado-Gunnison Basin a logical choice to implement the USGS WMA priorities of observing, delivering, assessing, predicting, and informing water resource conditions and decisions. The Delaware and Colorado-Gunnison Basins were ranked first in their hydrologic regions by the process developed here (Table 3).

**Fig. 6:**
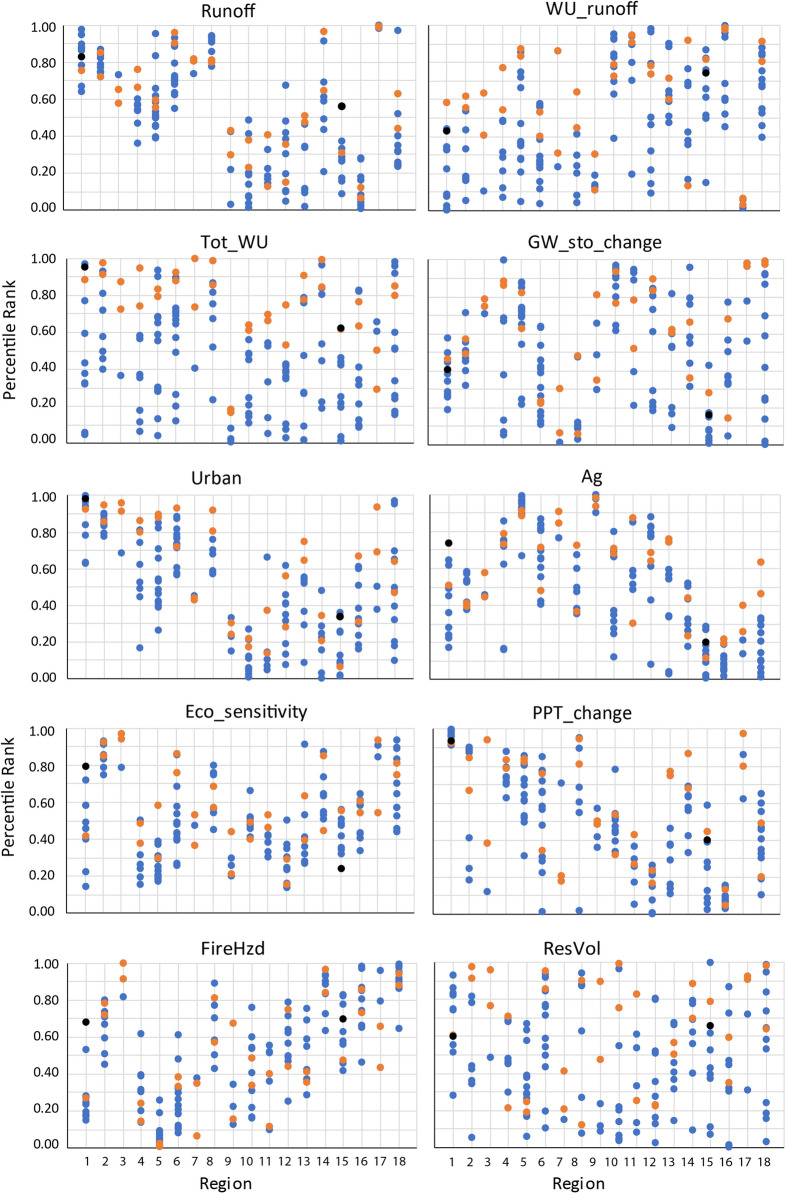
Distribution of candidate basins (all dots), top-two ranked basins (orange dots), and the Delaware River Basin and Colorado-Gunnison Basin (black dots for regions 1 and 15, respectively) by region for the 10 ranking variables (Variables are defined in Table 1, and regions are shown on Fig. 1)

The top-ranked basins can be compared to all candidate basins based on the 10 ranking variables (Fig. 6). The top-two basins in each region are identified by orange dots and the first two NGWOS basins selected, Delaware River Basin and Colorado-Gunnison Basin, are identified by the black dots in regions 1 and 15, respectively. These graphs indicate for a given basin which variables set it apart from other basins in the region and how it compares to the distribution of the variables nationally. As expected, the orange dots tend to be higher in percentile rank for the variables than are the blue dots (lower ranked basins) in each region. The Delaware River Basin is at the high end of the range nationally for *Tot_WU*, *Urban*, and *PPT_change*. Within Region 1, it is highest for *Ag*, *Eco_sensitivity*, and *FireHzd*, and second highest for *Urban* and *Tot_WU*. The Colorado-Gunnison Basin is midrange for most variables nationally but at the high end of the range within the Central Rockies for *Runoff*, *Tot_WU*, and *Urban*.

There is no independent test that we are aware of to measure the “success” of the ranking approach presented here. However, the approach prioritizes basins based on independent variables representing anthropogenic stressors of water resources and ecology. Thus, a comparison of the rankings to an independent index of watershed condition might indicate whether the objective of prioritizing basins facing water resource challenges has been met. We compared the rankings by region to the EPA’s Index of Catchment Integrity (ICI). Flotemersch et al. (2016) define watershed ‘integrity’ as “the capacity of a watershed to support and maintain the full range of ecological processes and functions essential to the sustainability of biodiversity and of the watershed resources and services provided to society”. Six sub-indices are incorporated into the ICI: hydrologic regulation, regulation of water chemistry, sediment regulation, hydrologic connectivity, temperature regulation, and habitat provision (Johnson et al. 2019). The “catchment” in the ICI means that the indices are computed for the immediate drainage area to each stream segment; the companion “Index of Watershed Integrity” is computed similarly but is accumulated for the full watershed for each stream segment; both are available from the EPA StreamCat Dataset (Hill et al. 2016).

**Fig. 7:**
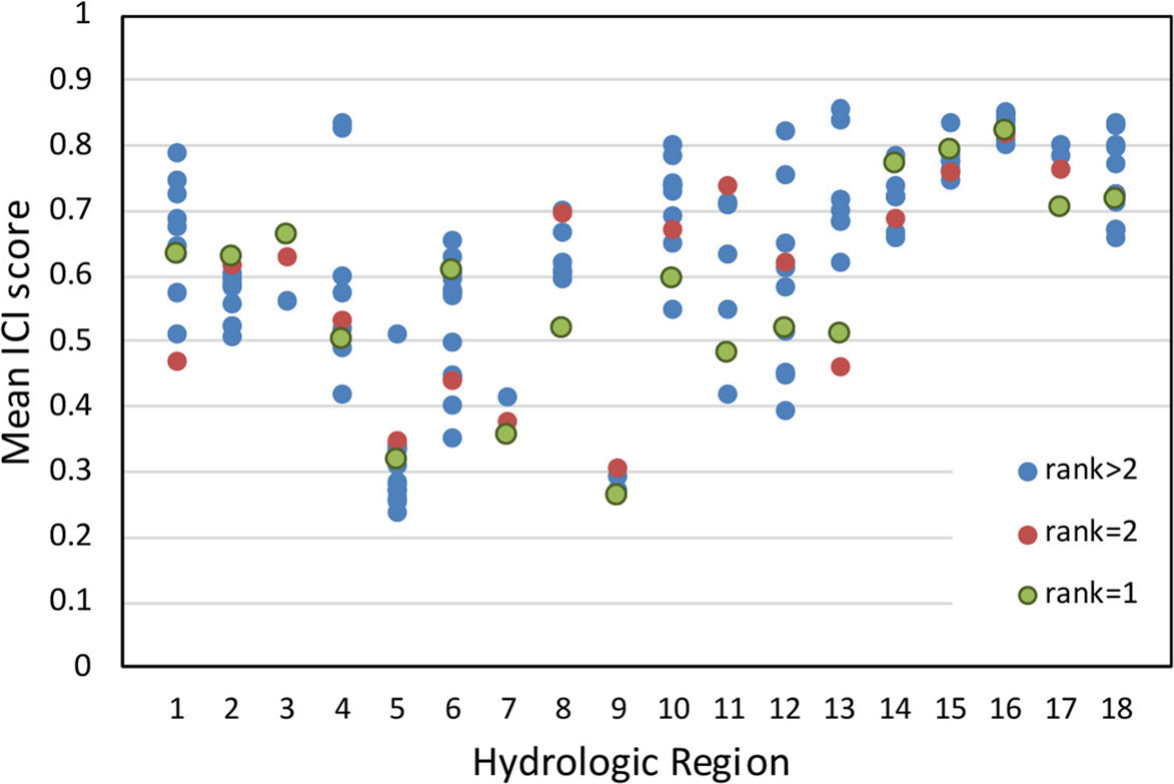
Comparison of ranking by hydrologic region to mean Index of Catchment Integrity (ICI) for all stream catchments in the region

Because the rankings within each hydrologic region incorporate anthropogenic stressors of water resources, we assume the higher ranked basins will have relatively low ICI scores. This outcome might indicate that the rankings are achieving our objective of identifying basins with high levels of anthropogenic stress on their water resources. The top and(or) second ranked basins are at the low end of the ICI score range in over half of the regions, especially in the central and western USA (Fig. 7). Conversely, in regions 2, 3, and 5, the top- and second-ranked basins have high ICI scores compared to other basins in the region. We note, however, that all of the basins in those three regions, and in several other regions, have ICI scores spanning a narrow range, meaning all of the basins have relatively similar condition indices. We also note that as a group, the top ranked basins span nearly the full range of ICI scores for the CONUS, which is a positive result of regionalizing the ranking. Not only does the regional framework mean that a wide range of hydrologic characteristics will be represented by the 10 studied basins but a wide range in watershed conditions also will be represented.

We recognize that there is no “best” approach to ranking basins for study based on their natural and anthropogenic characteristics. Any approach will reflect the choices of what variables to include and how those variables are manipulated and these choices will be influenced by the people making them and the objectives of the study. Furthermore, there is no objective way to test the outcome of this exercise in that “success” can only be measured after carrying out the studies, and no alternative outcomes of selection will occur. The ranking approach adopted here prioritizes the more stressed basins in terms of human development and water use within each hydrologic region. The choice to prioritize stressed basins was made by the BST to ensure that the study basins will have high societal relevance in that they are more likely to face critical water resource issues that affect more people than the lower ranked basins. This outcome could run counter to the objective of selecting basins that best represent the natural hydrologic conditions of each region to support national modeling objectives. The tradeoff is that the set of priority study basins will have high societal relevance but perhaps less power to refine and calibrate national-scale hydrologic models. However, many hydrologic models have focused on undisturbed natural settings rather than on heavily altered settings, thus, focusing resources on areas with altered settings could help us to improve the integration of economic and social drivers into physical modeling systems.

Establishing a regional framework based on hydrologic characteristics and distributing basins across the CONUS with no more than one basin per hydrologic region will help ensure that each basin represents a unique combination of important natural characteristics affecting the hydrologic cycle. Ranking within each region based on natural and anthropogenic factors such as land use, water use, runoff, water availability stresses, climate change, and ecosystem sensitivity should mean that basins selected for study represent important water resources and related environmental and socio/economic challenges for the nation.

## Electronic supplementary material

ESM 1 (XLSX 147 kb)
