# Correction to: Prioritizing river basins for intensive monitoring and assessment by the US Geological Survey

**DOI:** 10.1007/s10661-023-11173-1

**Published:** 2023-05-13

**Authors:** Peter C. Van Metre, Sharon Qi, Jeffrey Deacon, Cheryl Dieter, Jessica M. Driscoll, Michael Fienen, Terry Kenney, Patrick Lambert, David Lesmes, Christopher A. Mason, Anke Mueller-Solger, Marylynn Musgrove, Jaime Painter, Donald Rosenberry, Lori Sprague, Anthony J. Tesoriero, Lisamarie Windham-Myers, David Wolock

**Affiliations:** 1 US Geological Survey, Austin, TX USA; 2 US Geological Survey, Portland, OR USA; 3 US Geological Survey, Richmond, VA USA; 4 US Geological Survey, Baltimore, MD USA; 5 US Geological Survey, Lakewood, CO USA; 6 US Geological Survey, Madison, WI USA; 7 US Geological Survey, West Valley City, UT USA; 8 US Geological Survey, Reston, VA USA; 9 US Geological Survey, Sacramento, CA USA; 10 US Geological Survey, Norcross, GA USA; 11 US Geological Survey, Menlo Park, CA USA; 12 US Geological Survey, Lawrence, KS USA

**Correction to: Environ Monit Assess (2020) 192: 458** **https://doi.org/10.1007/s10661-020-08403-1**

The original version of this article unfortunately contained errors in Figures 4, 6, 7 and Tables S3, S5 and Table 3.

The Figures 4, 6, 7 and Tables S3, S5 and Table 3 were published erroneously.

The corrected Figures 4, 6, 7 and Tables S3, S5 and Table 3 are shown in the next page.

**Figure:**
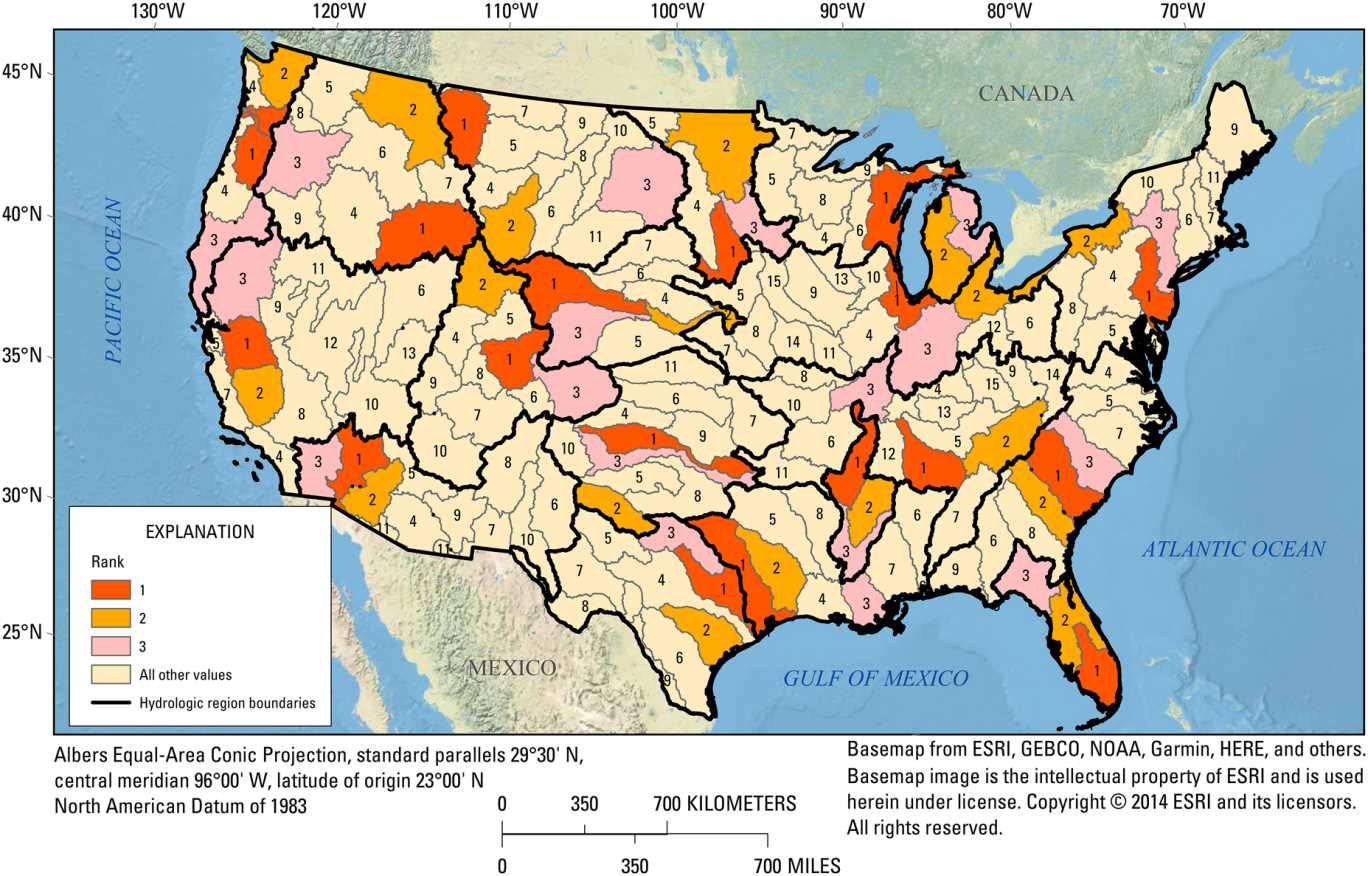

**Figure:**
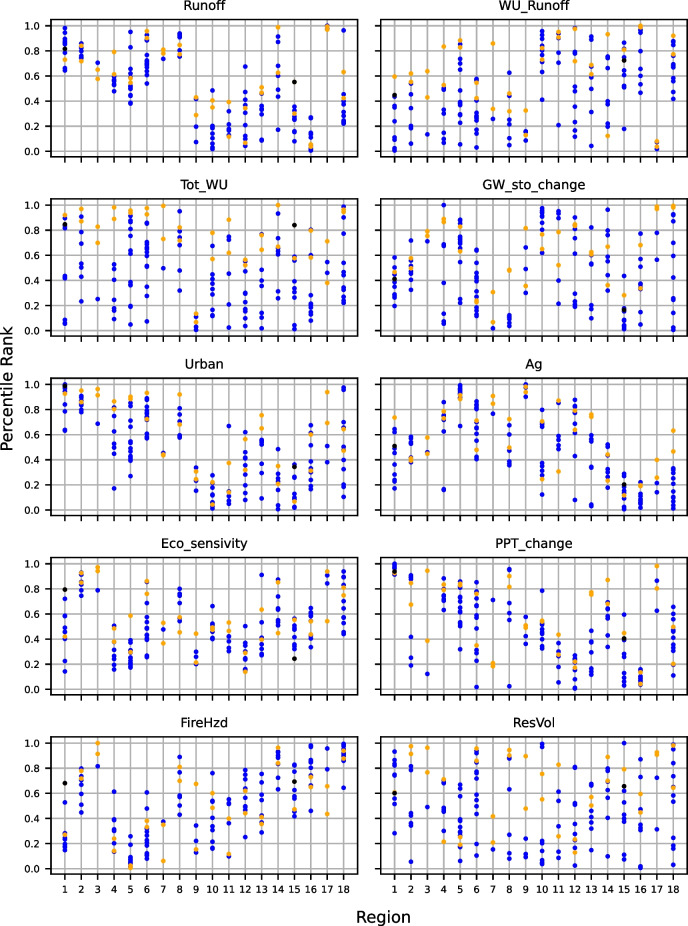

**Figure:**
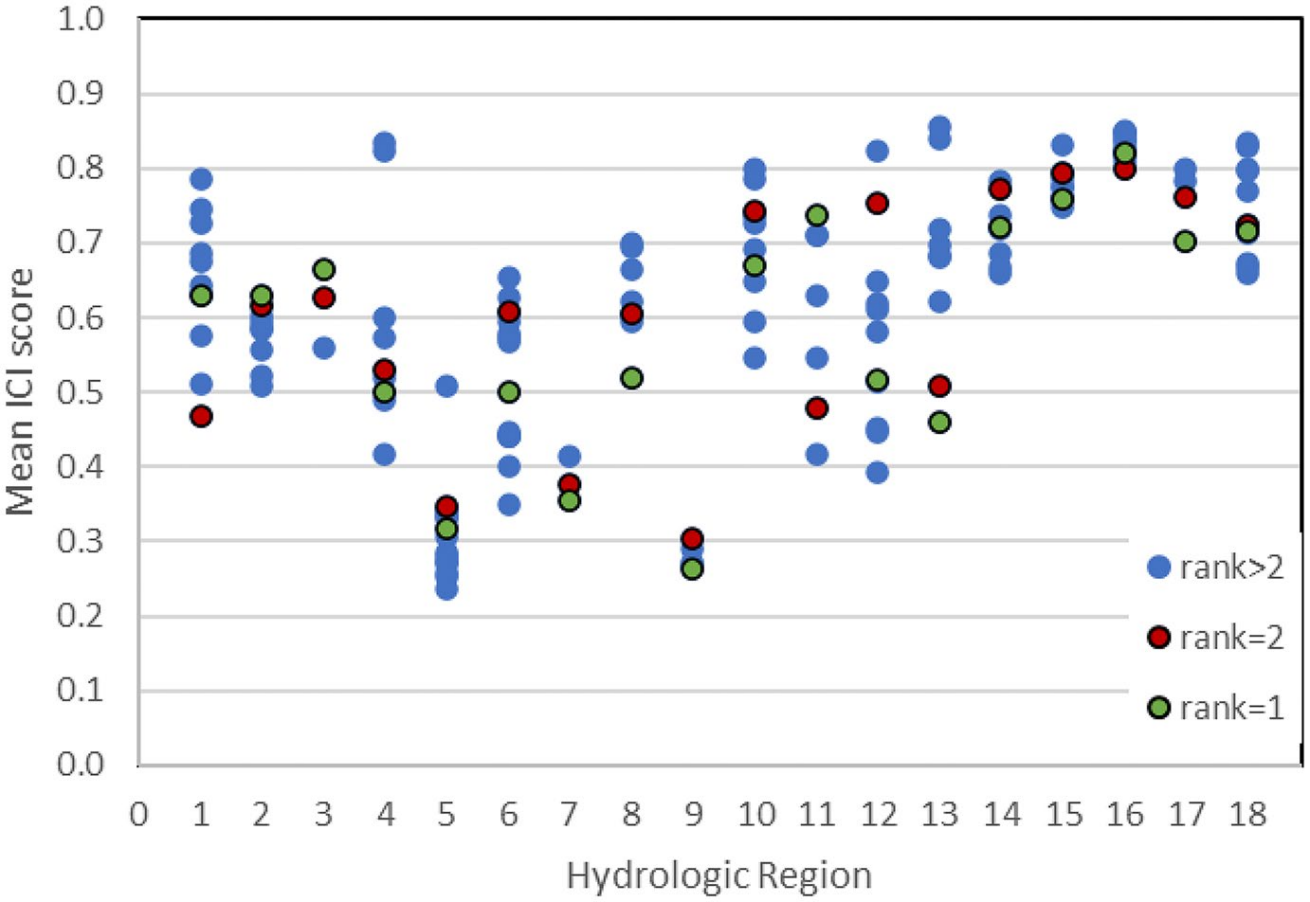

**Table:** **Table 3** Top-two candidate basins in each hydrologic region based on numerical ranking

REGION name	Basin ID	Basin name	Region#	Rank
Northeast	204	Delaware	1	1
	411	Lake Erie and Ontario	1	2
Atlantic Coast	305	Edisto–Santee	2	1
	306	Ogeechee–Savannah	2	2
Florida	309	Southern Florida	3	1
	308	Florida northcentral	3	2
Great Lakes	403	Western Lake Michigan	4	1
	405	Eastern Lake Michigan	4	2
Midwest	712	Upper Illinois	5	1
	409	Western Lake Erie	5	2
Tennessee–Missouri	603	Lower Tennessee	6	1
	601	Upper Tennessee	6	2
Mississippi Embayment	802	Lower Mississippi–St. Francis	7	1
	803	Lower Mississippi–Yazoo	7	2
Gulf Coast	1203	Trinity–San Jacinto	8	1
	1201	Sabine-Neches	8	2
Souris-Red-Rainy	1017	Missouri–Big Sioux	9	1
	902	Red	9	2
Northern High Plains	1003	Missouri–Marias	10	1
	1008	Big Horn	10	2
Central High Plains	1018	North Platte	11	1
	1020	Platte	11	2
Southern High Plains	1110	North Canadian	12	1
	1205	Brazos Headwaters	12	2
Texas	1207	Lower Brazos	13	1
	1210	Central Texas Coastal	13	2
Columbia–Snake	1704	Upper Snake	14	1
	1701	Kootenai–Pend Oreille–Spokane	14	2
Central Rockies	1401	Colorado-Gunnison	15	1
	1404	Great Divide–Upper Green	15	2
Southwest Desert	1503	Lower Colorado	16	1
	1507	Lower Gila	16	2
Pacific Northwest	1708	Willamette	17	1
	1711	Puget Sound	17	2
California-Nevada	1804	San Joaquin	18	1
	1803	Tulare-Buena Vista Lakes	18	2

## Supplementary Information

Below is the link to the electronic supplementary material.

Supplementary file1 (DOCX 17 kb)

Supplementary file2 (XLSX 147 kb)

