# Supplementary material for: Correction to: Prioritizing river basins for intensive monitoring and assessment by the US Geological Survey
Source: Environ Monit Assess. 2023 May 13;195(6):665. doi: 10.1007/s10661-023-11173-1 (PMC10181963; doi:10.1007/s10661-023-11173-1)
Supplement: Supplementary file 1 — Supplementary file1 (DOCX 17 kb) [file 10661_2023_11173_MOESM1_ESM.docx]

| Table 3 Top-two candidate basins in each hydrologic region based on numerical ranking | | |  |  |
| --- | --- | --- | --- | --- |
| REGION name | Basin ID | Basin name | Region# | Rank |
| Northeast | 204 | Delaware | 1 | 1 |
|  | 411 | Lake Erie and Ontario | 1 | 2 |
| Atlantic Coast | 305 | Edisto–Santee | 2 | 1 |
|  | 306 | Ogeechee–Savannah | 2 | 2 |
| Florida | 309 | Southern Florida | 3 | 1 |
|  | 308 | Florida northcentral | 3 | 2 |
| Great Lakes | 403 | Western Lake Michigan | 4 | 1 |
|  | 405 | Eastern Lake Michigan | 4 | 2 |
| Midwest | 712 | Upper Illinois | 5 | 1 |
|  | 409 | Western Lake Erie | 5 | 2 |
| Tennessee–Missouri | 603 | Lower Tennessee | 6 | 1 |
|  | 601 | Upper Tennessee | 6 | 2 |
| Mississippi Embayment | 802 | Lower Mississippi–St. Francis | 7 | 1 |
|  | 803 | Lower Mississippi–Yazoo | 7 | 2 |
| Gulf Coast | 1203 | Trinity–San Jacinto | 8 | 1 |
|  | 1201 | Sabine-Neches | 8 | 2 |
| Souris-Red-Rainy | 1017 | Missouri–Big Sioux | 9 | 1 |
|  | 902 | Red | 9 | 2 |
| Northern High Plains | 1003 | Missouri–Marias | 10 | 1 |
|  | 1008 | Big Horn | 10 | 2 |
| Central High Plains | 1018 | North Platte | 11 | 1 |
|  | 1020 | Platte | 11 | 2 |
| Southern High Plains | 1110 | North Canadian | 12 | 1 |
|  | 1205 | Brazos Headwaters | 12 | 2 |
| Texas | 1207 | Lower Brazos | 13 | 1 |
|  | 1210 | Central Texas Coastal | 13 | 2 |
| Columbia–Snake | 1704 | Upper Snake | 14 | 1 |
|  | 1701 | Kootenai–Pend Oreille–Spokane | 14 | 2 |
| Central Rockies | 1401 | Colorado-Gunnison | 15 | 1 |
|  | 1404 | Great Divide–Upper Green | 15 | 2 |
| Southwest Desert | 1503 | Lower Colorado | 16 | 1 |
|  | 1507 | Lower Gila | 16 | 2 |
| Pacific Northwest | 1708 | Willamette | 17 | 1 |
|  | 1711 | Puget Sound | 17 | 2 |
| California-Nevada | 1804 | San Joaquin | 18 | 1 |
|  | 1803 | Tulare-Buena Vista Lakes | 18 | 2 |
